# Rights and duties policy implementation in Chile: health‐care professionals’ perceptions

**DOI:** 10.1111/hex.12396

**Published:** 2015-08-18

**Authors:** Constanza R. Barrera, Camila P. Negrón, R. Mauricio Barría, Claudio A. Méndez

**Affiliations:** ^1^ Escuela de EnfermeríaFacultad de MedicinaUniversidad Austral de ChileValdiviaChile; ^2^ Instituto de EnfermeríaFacultad de MedicinaUniversidad Austral de ChileValdiviaChile; ^3^ Instituto de Salud PúblicaFacultad de MedicinaUniversidad Austral de ChileValdiviaChile

**Keywords:** health policy, health services research, patient rights

## Abstract

**Objective:**

To explore the perceptions of health professionals in an integrated network of public provision of health services regarding the implementation of the Law on Rights and Duties of People in Chile.

**Method:**

Qualitative descriptive study. A stratified qualitative sample of 53 professionals from five low complexity centres and one from a high complexity centre, all part of the integrated network of health services in Valdivia, Los Rios Region, Chile, were selected according to the criteria of an overall saturation of the explored dimensions. The information was gathered through a semi‐structured, in‐depth interview carried out after signing the informed consent. Data were analysed using an inductive approach of content analysis.

**Results:**

Three categories emerged from the interviews: conceptualization and knowledge, factors influencing the implementation and recommendations for strengthening the implementation, and seven subcategories. It was highlighted that health professionals in the health‐care network perceived difficulties in implementing the Law on rights and duties of patients. Among them were the lack of knowledge about the Law, poor exposure and a lack of resources for its implementation. They suggested adapting the infrastructure of the institution and offering training as recommendations to improve the implementation of the Law.

**Conclusions:**

There are hindering factors for the implementation of the Law related to organizational and professional gaps in the institutions providing health care.

## Introduction

Health systems are facing greater demands regarding health financing and provision of health services coverage, but also new kinds of patients who are aware of their rights and windows of opportunity for further public engagement.^1^ Some European Union member states have showed greater improvements in shifting their health systems to patients’ rights and duties by passing laws and ratifying multilateral conventions.^2^ Even though changes in the provision of health services originated by the design and implementation of laws on rights and duties of patients; these have been widely described from the patients’ perspective.^3^, ^4^, ^5^, ^6^, ^7^, ^8^, ^9^ The way health professionals perceive these changes regarding their key roles in the provision of health services remains without major evidence.

Chile has a mixed health system regarding its financing and provision of health services. The public provision, financed through general taxation, mandatory health tax and copayments, serves middle‐ and low‐income earners. Meanwhile, private provision, funded through mandatory health tax, copayments and out‐of‐pocket expenses that can be accessed by high‐income earners in the country.

The health system reform at the beginning of the 2000s, internationally known as the AUGE reform (acronym in Spanish for Universal Access with Explicit Health Guarantees) established changes in the organization of the health system which, although did not change its mixed structure, it did strengthen the stewardship of the system. This system separated the functions of public health from those related to the management of integrated health services networks, created the Superintendence of Health as an institution responsible for regulating the health system, and introduced an explicit health prioritization process based on explicit health guarantees.^10^, ^11^ Currently, there are 80 health problems and their interventions under the Regime of Explicit Health Guarantees.

On the other hand, the reform included a bill on the rights and duties of people regarding their health care. Although the project has entered discussion in the National Congress along with other projects of the reform, its promulgation and publication in the national Official Newspaper did not occur until April 2012. The Law introduced broad‐related rights from the protection in health care to the involvement of people in their health care.^12^ Furthermore, it established duties for patients with regard to respecting the internal rules of the institutions, staying informed on the functions of the institutions and providing respectful treatment to members of the health team.^12^

The rights and duties introduced by the Law are mandatory not only for public health‐care providers, but also for private ones. Therefore, health managers and health professionals are mandated to ensure the rights and duties independently of the public or private ownership and funding of health‐care providers.^12^ Moreover, both public and private providers are mandated to display the Patients’ Rights and Duties Chart in their facilities, and both are under the surveillance of the Superintendence of Health.

The World Health Report 2010 published by the World Health Organization, placed Chile as an example in the establishment of a system of guarantees that allows for moving towards universal health coverage.^13^ Thus, the Chilean health system is presented as an original case on how to provide universal health coverage under a law that establishes rights and duties for people. However, a year after the launch of the implementation phase there is no published empirical evidence on how health professionals perceive this implementation regarding the provision of health services in Chile.

This research aimed to explore the perceptions of health professionals in an integrated network of public provision of health services regarding the implementation of the Law on Rights and Duties of People in Chile.

## Methods

### Study design

A study of qualitative, descriptive and exploratory design was conducted. This type of design allows for the description and investigation of a scarcely understood phenomena, identifies or discovers meaningful units, and generates a new hypotheses of investigation^14^.This type of design was considered suitable for the study, given the absence of published empirical evidence regarding the perceptions of health professionals on the implementation of the Law on Rights and Duties of People in Chile and the public provision of health services in Chile.

### Study context

Los Rios Region is located in the far south of the country. Prior to 2007, it was part of Los Lagos Region, as a Province of Valdivia. Since that year, the new region was politically and administratively divided into the Provinces of Valdivia and Ranco. Considering the above, the data from the population census of 2002 estimated 356 396 inhabitants for the Province of Valdivia, which territorially corresponds to the current Los Ríos Region. The city of Valdivia is the regional capital, with a population of 140 559 inhabitants according to the same population census.^15^ The public health network of the city consists of a high complexity, teaching and assistance‐oriented hospital which is a reference centre for the region, and five family and community‐oriented centres for primary health care.

### Sampling

The sample population corresponded to health professionals from public health‐care providers who delivery health‐care services directly to patients. A first criterion for selecting professionals from public health‐care providers was due to the fact that these institutions manage a higher proportion of health professionals and deliver health care for almost the entire population of the city of Valdivia. The second criterion was selecting health professionals who provide health‐care services directly to the patients given its legal responsibility according to the Health Code of the Republic of Chile. Professionals in administrative‐only positions were excluded, because they do not take part in the direct provision of health services to patients. A stratified sample of 53 health professionals was considered.^16^ Participants were recruited until achieving the overall saturation of the dimensions and layers that were explored in the study.^17^ The professionals were personally contacted at their institutions by the research team. There was no refusal from the contacted professionals to participate in the study.

### Data collection

The qualitative technique of in‐depth and semi‐structured individual interviews was implemented.^18^ An interview protocol was designed to explore the various dimensions of the study. The topics addressed during the interview include; knowledge of the Law, factors that influenced its implementation and their recommendations on policy options to improve implementation. The interview protocol was subjected to a pilot test with professionals that had not been included in the final sample, as a quality criterion in qualitative investigations.^19^

Fieldwork was conducted between April and November 2013. Interviews lasted a minimum of 20 min and a maximum of 30 min and were conducted at the workplace of the respondents. The interviews were conducted in Spanish by trained interviewers, in order to obtain consistency and reduce the variation in the approach to the subjects presented to the respondents. After the informed consent process and the submission of the respective document, the interviews were carried out, recorded and transcribed verbatim. Once transcribed, and as a criterion of good quality regarding the report of qualitative inquiries investigations, they were sent to the respondents for comments and corrections.^19^

### Data analysis

The content analysis technique was used in its conventional approach.^20^, ^21^ This technique reduces the information through the definition of analysis units and coding trees. Once the units and their respective codes were established, subject categories and subcategories that emerged from the analysis of the talks were established. During the third stage, verbatim quotes from the interviews were selected. The quotes were translated into English and coded to protect anonymity and differentiate the workplace of the respondents: low complexity centres (LC) and high complexity centres (HC).

### Qualitative criteria rigour and quality

The quality of the research was supervised through the scientific criteria of credibility, dependability, confirmability and transferability.^22^, ^23^, ^24^ Credibility was achieved through triangulation of researchers and delivery of transcripts to the respondents. Dependence and confirmability were accomplished through the systematic description of the methodology used and reflexivity in conducting the analysis. Finally, transferability was achieved through structural, organizational and legal homogeneity of the public provision of the Chilean Health System, which allows to transfer part of the findings to other regional and community care networks that meet the different characteristics of the studied network.

### Ethical considerations

The research protocol was approved by the Ethics Research Committee of the Health Service of Valdivia (Ref: No. 044‐2013). Also, the respective authorizations were requested from the administrative authorities of the institutions providing health services. Finally, an informed consent document was given to the respondent that stated the goal of the research, as well as how they would be participating on a volunteer basis and acknowledged the freedom to leave the investigation without any explanation.

## Results

**Figure 1:**
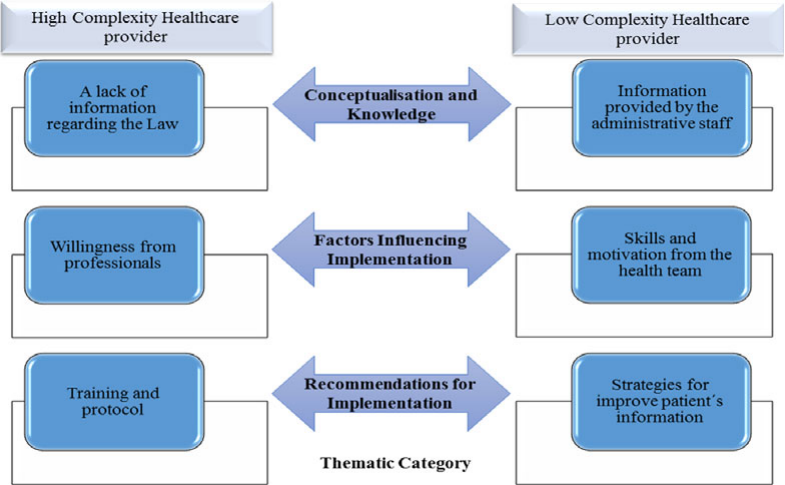
Health professionals’ perceptions on rights and duties implementation by healthcare provider complexity.

According to the level of complexity of the institution providing health services, 54.7% of the professionals work at low complexity centres and 45.3% at high complexity centres. According to gender, 67.9% of them were professional women (Table 1). Thematic categories and subcategories were considered, which emerged from the analysis of the interviews to describe the results (Table 2). In Fig. 1, the broader results are exhibits according the thematic categories and type of health‐care provider complexity.

**Table 1:** Qualitative sample features of health‐care pro‐fessionals from the public integrated health‐care delivery network from the city of Valdivia, Los Ríos Region, April–November 2013

Variable	Frequency	
	*n*	%
Sex		
Female	37	67.9
Male	16	32.1
Age		
20–30	13	24.5
31–40	24	45.2
41–50	9	17
51–60	5	9.4
61–70	2	3.9
Profession		
Registered nurse	13	24.5
Physician	10	19
Registered midwife	8	15.1
Nutritionist	5	9.4
Kinesiologist	7	13.2
Dentist	4	7.5
Social worker	4	7.5
Occupational therapist	2	3.8
Years of service		
01–10	34	64.1
11–20	9	17
21–30	6	11.3
31–40	4	7.6
Provider complexity level		
High complexity care	29	54.7
Low complexity care	24	45.3

**Table 2:** Thematic categories and subcategories that emerged from the content analysis of discourses of health‐care professionals from the public integrated health‐care delivery network from the city of Valdivia, Los Ríos Region, April–November 2013

Category	Subcategory
Conceptualisation and knowledge	General knowledge
	Training
Factors influencing implementation	Facilitating factors
	Hindering factors
Recommendations for implementation	Divulgation and training
	Technical foundations of design
	Infraestructure of institutions

### Conceptualization and knowledge

The professionals at low complexity centres report that knowledge regarding the Law was acquired in their workplace through the information provided by administrative staff. The best‐known rights were those related to respect and good treatment towards the user, providing timely care and information on issues related to health care. In the case of duties, the compliance of the regulations of the health centre, providing truthful information during care and not to abuse workers was mentioned.

Professionals at high complexity centres reported that the information they were given at work was scarce, there was no training, and providing information was emphasized only at the beginning of the implementation of the Law. They mentioned that the most important rights to maintain were; privacy, to keep the user informed, and confidentiality of the medical records. Among the duties, they know the obligation to comply with the internal regulations of the institution, relating it to reported conflicts with the families of patients.

…Perhaps only ignorance, from my part and perhaps many of us are not very informed and if we were asked we might not be able to give a clear response…

[LC‐19]

…I saw a poster, and I have heard of it on the news…but I don't know what it is about… I mean, the rights and duties of patients but I don't know in detail….

[HC‐52]

Professionals at low complexity centres showed ignorance about how the implementation stage is being assessed. Similarly, professionals differ regarding the organizational body responsible for evaluation: Information Office, Complaints and Suggestions (OIRS) for the patients, through the User Satisfaction Survey or undercover. In the case of professionals at high complexity centres, they perceive that the implementation of the Law is not being monitored. Also, they say they have not been subject of a compliance assessment.

…I don't know, because here it has always been fulfilled, the patients are kept informed… at least in my area I report what the patient has, the privacy issue is maintained and I think it happens that way, but I don't know if at supervising level the Health Service might be implementing some kind of supervision, so to speak…

[LC‐21]

…I think it was the same for accreditation at least here, because everyone focused just on that and nothing else…but more at national level. I don't know, I think it because of misinformation, because it is a law and not a protocol, like infection procedures or that sort of thing, I don′t know either if there will be any penalty…

[HC‐01]

The perceptions of the professionals at low complexity centres, as well as those from professionals at high complexity centres, agree regarding the absence of an information strategy for the implementation of the Law. Moreover, they perceived a lack of assessment on the implementation and enforcement of the Law.

### Factors influencing the implementation

For the professionals at low complexity centres, the factor that facilitates the implementation has been the set of skills, motivation and commitment of the health team to educate both workers and users regarding the relevance of the Law. However, for the professionals at high complexity centres, the organizational and political factors that facilitated the implementation of the Law were scarce, highlighting only the provision of information by the staff and the willingness of the professionals to assist the patients and their families.

…At least where I work the willingness of people… the staff is clear about it and we sort of put an effort into it. It is not that we're talking all day about rights and duties, but…I do not know…

[LC‐22]

…For them to facilitate…well, knowledge…and the will to enforce things…because like I said things are basic common sense…

[HC‐08]

The hindering factors identified by the professionals at low complexity centres were the general ignorance on the content of the Law by patients, professionals and health technicians. Although professionals at the high complexity centres also perceive that the limited knowledge of the Law is an obstacle to its implementation, they think that the hospital accreditation process to which the institution was subjected also contributed as an obstacle to its implementation. This is because it was given institutional priority over the implementation of the Law.

…The lack of exposure, it might little information. Although it is in the waiting room and somehow people might have heard of it, but… you have to read it, but there are people who cannot read. It is as if it was only depending on that, whoever is interested in finding out is going to read it but perhaps more is needed…

[LC‐28]

…I think that yes sometimes they were giving talks, posters were put up, but I think that lasted about a month and then no longer, I saw more people worried about accreditation more than anything, about studying the different parameters it has, but the relationship with the rest of the patients seems to be left aside…

[HC‐14]

The professionals perceive the design of the Law as a highly centralized stage, which did not consider the opinion of those who would be responsible for implementing it. This characteristic is attributed to the design stage and has caused the main obstacles to its implementation.

### Recommendations for strengthening the implementation

To improve the implementation of the Law, professionals at the low complexity centre identify as policy options to develop strategies to improve the delivery of information to patients, as well as to educate people through the training of community leaders so they impart the Law in a way that is comprehensible to the people. In the case of professionals at high complexity centres, a policy option should be directed to the training of health professionals on matters of standardization processes through protocols to implement the Law.

…I think that to inform…inform the people, maybe to have more community meetings, maybe channel it through leaders…

[LC‐43]

…It's super important that training is continuous, well communicated. It is something every professional must know in order to work in health, so maybe it could be something to be included in the training, when they are students. And everything should be standardized, so that we all speak the same language…

[HC‐06]

Professionals at both low and high complexity centres perceive the need to deepen the fundamentals behind the design and implementation of the Law. For them, the fact that a Law had been designed focused exclusively on the rights of patients, without considering a similar number of duties, is perceived as a barrier to improve the provision of health services. Moreover, they perceive that to deepen its concept would be an advantage to move towards a greater commitment to the fulfilment and dissemination of the Law to patients.

…We should empower it a bit more, get to know it better, it would be super important to know how this Law was created, why was it created. What was the need for a new bill of rights and duties from the authority and not from the community…

[LC‐27]

…To make a more clear program regarding what is intended with admission…because like I said…I don′t know, posters were disseminated and put up and it was all left there…now what?… I don′t know, that could be clarified a bit more, I mean, I know they can sue you if you don′t comply with something…but if you don′t comply and the patient does not take action because I don′t know…he doesn't know…its family is from the countryside or whatever…what happens if you don′t comply? Nothing happens…?

[HC‐02]

On the other hand, professionals at the low complexity centres recommend improving structural aspects of institutions that act as barriers to the implementation of the Law. They recommend investing in infrastructure and increasing the number and size of the rooms for patient care. Similarly, practitioners at the high complexity centres recommend expanding the physical space of complex services with high demand to guarantee privacy, respect and dignity of patients.

…We feel affected by the user, because he demands, we know that the center is under a process of normalization and that there will be in infrastructure, we are all tight, there is no box. I will I see you here today, there another day and the patient gets angry because he doesn′t find you and there he goes, letting his anger out, I don't know…

[LC‐18]

I think one has to know how to work with what you have. But a small caring facility is not fit to serve so many people. I think they have to start by sorting things out at all levels. Because we try to comply with what we have but overall it should change…

[HC‐12]

For professionals in both levels of complexity, the changes that are needed to improve the implementation of the Law, as well as its purpose, must include structural changes in the institutions that provide health services, as well as working the alignment with professionals regarding the contents of the Law.

## Discussion

The findings of this investigation allow establishing the relevance of the perceptions of professionals who provide health services on the implementation of laws aimed at strengthening the Rights and Duties of patients. Moreover, they help to identify factors that contribute to overcome the incompatibility of moving forward on rights of patients, for which part of the health system is not prepared to meet.

The experience of Chile shows the complexity of moving towards the implementation of Rights and Duties of patients under a double logic of providing health services. Firstly, the workforce must face the demand of pathologies with Explicit Health Guarantees, and secondly other pathologies whose provision is not subjected to that scheme. Even though several investigations on the implementation of the health reform have shown how the gaps between design and implementation capacity of the institutions and the workforce have acted as obstacles to reach the objectives of the health system.^25^, ^26^ This is apparent in the case of the implementation of the Law on Rights and Duties of patients as they are again placed as a central aspect.

An interesting finding was related to the perception of professionals on the technical discussion behind the content of the Law. Although the results of the investigation were part of a widely debated and divulgated reform proposal, they could be explained by the time elapsed between the project and the enactment of the Law. In the same sense, this can be noted in the perception of professionals at different levels of complexity regarding the few duties that the Law request from patients. Even if the studies on perceptions of health professionals regarding laws on Rights and Duties of patients are scarce, their main finding is the ignorance of the professionals on the rights they must safeguard in their patients.^27^, ^28^

Even if the implications for and against implementing duties on patients of publicly funded health systems has been discussed,^29^ in the case of the Chilean experience, rights and duties include providers with public and private ownership and, therefore, funding. Even though this feature makes the Chilean experience more comprehensive at implementing patients’ rights and duties, the obtained results pose the difficulties faced by the public provision of health services, both structurally and organizationally to enforce not only duties but also rights. In this regard, emphasis has been placed on how the policies of institutions, equipment, supplies, work environment and professional deficits affect the implementation of the rights of patients.^30^ Nevertheless, in the Chilean case, a top‐down policy approach could explain why professionals perceived a lack of a thorough implementation process. In fact, the first inspection of the Superintendence of Health regarding the compliance of the Law reported a significant number of public providers that do not have internal regulations for the compliance of the Law.^31^ As a path for overcoming these shortages, the key role of health professionals have been identified along patient's awareness.^32^, ^33^

Although the reported results are limited as they represent the perceptions of professionals of an integrated network of health services in a medium‐sized town in southern Chile, having a design process of public policies at a country level, which is centralized and implemented under an organizational and administrative structure in health and is being carried out in all regions, allows transferability of results.

In conclusion, the policy implementation process has shown gaps between design and implementation stages from the health‐care reform. Thus, there are hindering factors for the implementation of the Law related to organizational and professional gaps in the institutions providing health care. By surpassing the professional gaps, it is also an opportunity for strengthening professionalism in a scenario to further patient's rights and duties. It has been argued that social factors such as increased attention to health‐care issues, changes in philosophy of care for patients and rapid changes in management of care are influencing the current changes on professionalism.^34^, ^35^ In the Chilean case, it seems these factors should be a matter of concern as professionals perceived a high concentration in patient's rights instead of duties.

Overcoming the implementation gaps will require moving towards policy options focused on bringing the divide between health professional's education and health system performance on patient's rights and duties. There will be also a priority moving towards a comprehensive design of strategies for supporting health professionals and patients in the implementation phase. However, further research is needed regarding monitoring the enforcement of the Law, as well as the relationship of the implementation of the Law with better results linked to the accomplishment of the non‐medical expectations of people either in public or private health‐care providers.

## Conflict of interest

The authors do not have any conflict of interest.
